# Nutritional status of indigenous children: findings from the First National Survey of Indigenous People’s Health and Nutrition in Brazil

**DOI:** 10.1186/1475-9276-12-23

**Published:** 2013-04-03

**Authors:** Bernardo L Horta, Ricardo Ventura Santos, James R Welch, Andrey M Cardoso, Janaína Vieira dos Santos, Ana Marlúcia Oliveira Assis, Pedro CI Lira, Carlos EA Coimbra Jr

**Affiliations:** 1 Programa de Pós-Graduação em Epidemiologia, Universidade Federal de Pelotas, Rua Marechal Deodoro 1160, Pelotas, RS, 96020-220, Brazil; 2 Escola Nacional de Saúde Pública, Fundação Oswaldo Cruz, Rua Leopoldo Bulhões 1480, Rio de Janeiro, RJ, 21041-210, Brazil; 3 Departamento de Antropologia, Museu Nacional, Universidade Federal do Rio de Janeiro, Quinta da Boa Vista s/n, Rio de Janeiro, RJ, 20940-040, Brazil; 4 Escola de Nutrição, Universidade Federal da Bahia, Rua Araújo Pinho 32, Salvador, BA, 40110-150, Brazil; 5 Departamento de Nutrição, Universidade Federal de Pernambuco, Avenida Professor Moraes Rego 1235, Recife, PE, 50670-901, Brazil

**Keywords:** Brazil, Indigenous peoples, Health surveys, Nutrition surveys, Health status indicators, Epidemiologic measurements, Child health, Nutritional status

## Abstract

**Introduction:**

The prevalence of undernutrition, which is closely associated with socioeconomic and sanitation conditions, is often higher among indigenous than non-indigenous children in many countries. In Brazil, in spite of overall reductions in the prevalence of undernutrition in recent decades, the nutritional situation of indigenous children remains worrying. The First National Survey of Indigenous People’s Health and Nutrition in Brazil, conducted in 2008–2009, was the first study to evaluate a nationwide representative sample of indigenous peoples. This paper presents findings from this study on the nutritional status of indigenous children < 5 years of age in Brazil.

**Methods:**

A multi-stage sampling was employed to obtain a representative sample of the indigenous population residing in villages in four Brazilian regions (North, Northeast, Central-West, and Southeast/South). Initially, a stratified probabilistic sampling was carried out for indigenous villages located in these regions. Households in sampled villages were selected by census or systematic sampling depending on the village population. The survey evaluated the health and nutritional status of children < 5 years, in addition to interviewing mothers or caretakers.

**Results:**

Height and weight measurements were taken of 6,050 and 6,075 children, respectively. Prevalence rates of stunting, underweight, and wasting were 25.7%, 5.9%, and 1.3%, respectively. Even after controlling for confounding, the prevalence rates of underweight and stunting were higher among children in the North region, in low socioeconomic status households, in households with poorer sanitary conditions, with anemic mothers, with low birthweight, and who were hospitalized during the prior 6 months. A protective effect of breastfeeding for underweight was observed for children under 12 months.

**Conclusions:**

The elevated rate of stunting observed in indigenous children approximates that of non-indigenous Brazilians four decades ago, before major health reforms greatly reduced its occurrence nationwide. Prevalence rates of undernutrition were associated with socioeconomic variables including income, household goods, schooling, and access to sanitation services, among other variables. Providing important baseline data for future comparison, these findings further suggest the relevance of social, economic, and environmental factors at different scales (local, regional, and national) for the nutritional status of indigenous peoples.

## Introduction

Nutritional status during childhood is a multidimensional condition strongly influenced by a large set of sociodemographic, economic, environmental, and biological factors, with prevalence rates of undernutrition being higher among children living under unfavorable conditions [1,2]. Among indigenous peoples, prevalence rates of undernutrition and many other indicators point to poorer health status, at times reaching alarming levels, as compared to non-indigenous peoples in the same regions. Helping to explain these health disparities are comparative analyses indicating that indigenous peoples are among the most politically and socioeconomically marginalized segments of society in the many countries in which they are present [3-5].

In specific indigenous ethnic groups in the Andean and Amazonian regions of South America, up to two thirds of school-aged children are undernourished [6-12]. In Brazil, despite major advances in public health indicators having occurred in recent decades for the general population, including a marked reduction in the overall prevalence of all forms of undernutrition in children [13], the nutritional situation of indigenous children remains worrying. As reported in recent nutritional assessments of different local indigenous communities in the country, chronic undernutrition is among the principal health problems, often affecting the growth of up to half of all children [14-19].

Within Brazil, indigenous children in the North region (which largely coincides with the Amazon region) present the highest prevalence rates of low height-for-age as compared to the country’s other regions [20,21]. Highlighting the comprehensive influence of socioeconomic and environmental conditions on child nutrition, indigenous children in the North also present higher prevalence rates of other nutritional deficit indicators, such as anemia, and are exposed to poorer sanitation and socioeconomic conditions (e.g., access to clean drinking water and precarious living conditions) [20], which are strictly associated with chronic undernutrition. The growing body of literature on nutritional profiles of indigenous populations in the Amazon region show the frequencies of chronic undernutrition in children < 5 years of age to vary markedly from 10-20% to 50-60% in association with diverse sociocultural, economic, and environmental variables [14,22-24]. Recent diachronic studies in the Amazon demonstrate that improved socioeconomic conditions and access to sanitation and health services resulted in substantial reductions over time in the prevalence rates of chronic undernutrition among indigenous children. For example, among the Amazonian Suruí, the prevalence of undernutrition in children < 5 years of age decreased from 46.3% to 26.7% in a period of less than two decades [15].

Undernutrition in childhood increases mortality rates and disease burden [1]. This observation is consistent with the findings of recent comparative analyses of health levels pointing to higher mortality and morbidity rates among indigenous peoples compared to the rest of the population in Latin America [3,4,25]. The same pattern was also observed in Brazil where, for example, infant mortality rates are much higher in indigenous populations than in the general population [26-28].

Undernutrition in the first years of life may also have long-term consequences on the development of chronic diseases and human capacities. Victora et al. [29] observed that low birthweight and chronic undernutrition in childhood were associated with increased blood pressure, blood glucose, total cholesterol, and mental illness in adulthood. On the other hand, it has also been suggested that rapid weight gain in the first years of life may reverse the long-term consequences of intrauterine growth restriction. For example, among Brazilian infants born small for gestational age (a proxy of intrauterine growth restriction), rapid weight gain in the first two years of life is related to higher achieved schooling in early adulthood [30] and higher birthweight in the next generation [31]. Therefore, promotion of weight gain in the first years of life may have positive long-term and intergenerational consequences [32,33].

Although child undernutrition rates and their associated factors have been studied for the general non-indigenous population and specific localized indigenous populations in Brazil, no previous study has addressed the subject for indigenous peoples at the national scale. The First National Survey of Indigenous People’s Health and Nutrition in Brazil (henceforth, “National Survey”), conducted in 2008–2009, was the first study to evaluate a nationwide representative sample of indigenous peoples living in villages throughout the country. With a final study population of 6,128 children from 113 villages, it included the most comprehensive investigation yet conducted on the nutritional situation of indigenous children in Brazil and one of the most extensive and detailed ever carried out in Latin America. The present article assesses the nutritional status of indigenous children < 5 years of age in Brazil.

## Methods

The National Survey sought to assess the health and nutritional status of indigenous children < 5 years of age and women 14 to 49 years of age in Brazil [20]. A multi-stage sampling was employed to obtain a representative sample of the country’s official geopolitical regions North, Northeast, Central-West, and South/Southeast (the South and Southeast regions were joined for the purposes of the National Survey). These regional strata are differentiated by diverse environmental, economic, social, and political factors [34], including distinct histories of demographic and economic expansion. Whereas the coastal Northeast and South/Southeast regions were the first to be colonized by Europeans, encroachment of indigenous lands to the west and north, including the Amazon region, generally occurred more recently under comparatively favorable policies for recognizing indigenous lands.

Initially, a stratified probabilistic sampling was carried out for indigenous villages located in these four regions. The basis for this sample was a list of indigenous villages provided in 2008 by the Brazilian National Health Foundation (Fundação Nacional de Saúde – FUNASA). From the original list containing 3,995 villages, 1,227 (30.7%) were excluded for the purposes of selection because they were identified by FUNASA as vacated (“desaldeadas”), deactivated, or having less than 31 inhabitants. The sample size for each region was estimated based on the size of its target population, a prevalence of 50% for all disease outcomes, a relative precision of 5%, and a confidence level of 95%, according to the methodology proposed by Lemeshow [35]. Based on the calculated sample size for each region, Sequential Poisson Sampling criteria were used to select villages [36]. The final sample included 123 villages distributed by region as follows: 65 (North), 14 (Central-West), 23 (Northeast), and 21 (South/Southeast).

Subsequently, two strategies were used to sample indigenous households in the sampled villages (census and sample). A census was carried out in villages with populations of children < 5 years and women from 14 to 49 years of age ≤ 150. In villages with populations of women and children greater than 150, households were selected by systematic sampling. Further details on the study methodology have been published previously [20].

Workshops were held to train multidisciplinary field teams in research and anthropometric measurement procedures [20]. To guarantee the precision and reliability of field measurements, designated anthropometrists participated in training exercises to standardize their use of equipment and calculate within and between observer variability of height and weight measurements. The standard deviation of replicate measurements between observers and within observers was less than 5 mm [37].

In selected households, several questionnaires (domicile, mother, child) were applied in participating households. Questions in Portuguese addressed sociodemographic conditions, sanitation, domestic economy, access to health services, maternal characteristics, infant feeding, and morbidity [20]. The mothers or caretakers of all children < 5 years of age were interviewed. Children whose mothers self-identified as indigenous and children who were identified as indigenous by a caretaker or non-indigenous mother were included in the study. Local indigenous translators (often indigenous health agents or primary education teachers) were used for interviews with non-Portuguese speakers.

Children were weighed with a portable digital scale (seca model 872, Hamburg, Germany) to the nearest 100 g, wearing minimal clothing and barefoot. This scale has a function allowing children ≤ 24 months to be weighed while being held by an adult. Standing height was measured with an AlturaExata portable anthropometer (Belo Horizonte, Brazil) and recorded to the nearest 0.1 cm. This anthropometer was also used to measure recumbent length for children ≤ 24 months of age. Previously trained and standardized field researchers carried out anthropometric measurements. Basic birth data, including sex, birthdate, and birth weight, were obtained from local FUNASA healthcare records, participants’ personal documents (identification cards, birth certificates, and child health cards), or informed by interviewees.

Weight-for-age, height-for-age, and weight-for-height z-scores were estimated using the 2006 WHO growth standards, widely considered effective standardized public health indicators of child nutritional status worldwide [38,39]. Stunting (low height-for-age), underweight (low weight-for-age), and wasting (low weight-for-height) were defined by using the −2 z-score cut-off-point. Anthropometric indicators were interpreted using WHO growth reference curves, derived from multicentric samples of children raised under ideal environmental conditions, based on the operational assumption that they are appropriate for evaluating children worldwide, independent of ethnicity, socioeconomic condition, or diet growth references [40].

Following WHO guidelines [41], mothers whose hemoglobin concentrations were lower than 7.0 g/dL were considered to present severe anemia and those with concentrations from 7 to 11.99 g/dL were considered to present moderate anemia.

A household goods index was calculated using the first component of a principal component analysis for 19 durable goods (eigenvalue of 3.56, accounting for 19% of the total variability in the dataset) [20]. Standing out in this first component were television set, refrigerator and/or freezer, VCR and/or DVD player, stove, telephone, and satellite dish. The household index was the sum of the products of the quantity of each item multiplied by the contribution of each in the principal component analysis. Households were then classified in terciles based on the combined distribution, considering the four regions.

In the initial data analysis, prevalence rates were calculated for stunting, underweight, and wasting according to independent variables (region, demographic and socioeconomic variables, characteristics of the household environment, and maternal and child characteristics). Chi-square tests for linear trend and heterogeneity were used to evaluate differences in proportions.

In the multivariate analysis, Poisson regression with robust adjustment of the variance for dichotomous outcomes was used [42]. Estimates were corrected for the complex sampling design of the study. The variables were entered according to a hierarchical model. Accordingly, distal variables (region, child’s age, and sex) were the first to enter the model, followed by socioeconomic variables (household goods index, presence of regular income from salaries or social programs, and maternal schooling) in the second level. The third hierarchical level comprised variables that assessed household characteristics (source of drinking water, location used to defecate, and presence of trash collection in the village). The fourth and fifth most proximal levels encompassed maternal variables (age and anemia) and child variables (birthweight, reported hospitalization during the prior 12 months, and infant feeding), respectively. Estimates were adjusted for variables located in the same or higher hierarchical levels. Estimates of the effect of backward selection were used at each level and variables with p-values > 0.20 at their hierarchical level were excluded from the model [43].

The study protocol was approved by the National Ethics Committee (Comissão Nacional de Ética em Pesquisa – CONEP) and the National Indian Foundation (Fundação Nacional do Índio – FUNAI). Before initiating interviews in a given village, a meeting was held with community leaders to obtain permission to conduct the study. To the extent possible, these meetings were held in public and formulated according to local protocols for community decision-making. In addition to describing the objectives and procedures of the study, a collective informed consent form approved by CONEP was presented in detail. If consent was granted, one or more community leaders were asked to sign the consent form.

## Results

Of 123 villages selected for study, data were obtained for 113 (91.9%). Non-investigation of 10 villages was due to refusal, lack of access, cost, and loss of data. Of 5,674 indigenous households planned for investigation, 5,305 (93.5%) were interviewed. The principal reason for non-inclusion of households was absence at the time of research (5.9%). Of planned individual interviews regarding children, 6,128 (93.1%) were realized. Additionally, height and weight measurements were taken for 6,050 and 6,075 children, respectively, which correspond to 98.2% and 98.6% of the total sample investigated. Because the proportion of sampled children not evaluated was low, it is unlikely that the National Survey is susceptible to sample selection bias.

With respect to nutritional status, prevalence rates of underweight, stunting, and wasting were 5.9% (n = 6055), 25.7% (n = 6011), and 1.3% (n = 6017), respectively. Mean z-scores for weight-for-age, height-for-age, and weight-for-height were −0.48, −1.32, and 0.38, respectively.

Table 1 shows that the prevalence rates of underweight and stunting were highest among indigenous children in the North region and were slightly lower among girls nationally. Whereas child age was positively related to the prevalence of stunting, the association was negative for wasting.

**Table 1:** Prevalence rates of underweight, stunting, and wasting in indigenous children < 60 months by region, sex, and age, First National Survey of Indigenous People’s Health and Nutrition, Brazil, 2008-2009

	**N**^**†**^	**Underweight**		**Stunting**		**Wasting**	
		**Prevalence**	**PR**	**Prevalence**	**PR**	**Prevalence**	**PR**
		**(%)**	**(CI 95%)**	**(%)**	**(CI 95%)**	**(%)**	**(CI 95%)**
**Region**			p < 0.001 *		p < 0.001 *		p = 0.22 *
North	2558	11.4	2.87	40.8	1.83	1.7	1.50
			(1.61-5.11)		(1.21-2.77)		(0.61-3.68)
Central-West	1283	5.0	1.26	27.6	1.24	0.9	0.80
			(0.69-2.31)	13	(0.77-1.97)	1.	(0.29-2.20)
Northeast	1339	4.1	1.02		0.62		1.17
			(0.58-1.81)	.9	(0.38-1.01)	4	(0.43-3.23)
Southeast/South	875	4.0	1.00	22.3	1.00	1.2	1.00
			(reference)		(reference)		(reference)
**Sex**			p = 0.04 *		p = 0.03 *		p = 0.17 *
Male	3104	6.5	1.00	27.0	1.00	1.5	1.00
			(reference)		(reference)		(reference)
Female	2951	5.3	0.81	24.3	0.90	1.1	0.70
			(0.66-0.99)		(0.82-0.99)		(0.44-1.12)
**Child’s age (months)**			p = 0.06 **		p < 0.001 **		p < 0.001 **
0 – 5	644	4.0	1.00	9.2	1.00	2.7	1.00
			(reference)		(reference)		(reference)
6 – 11	675	7.3	1.83	14.9	1.63	2.6	0.96
			(1.03-3.26)		(1.15-2.31)		(0.45-2.05)
12 – 23	1201	7.5	1.88	31.5	3.43	2.0	0.73
			(1.11-3.16)		(2.58-4.57)		(0.35-1.52)
24 – 35	1175	6.1	1.52	32.7	3.57	0.4	0.16
			(0.88-2.63)		(2.62-4.85)		(0.05-0.46)
36 – 47	1243	5.1	1.27	28.3	3.09	0.7	0.24
			(0.70-2.30)		(2.24-4.27)		(0.08-0.72)
48 – 59	1117	5.1	1.28	25.2	2.75	0.6	0.22
			(0.77-2.15)		(1.94-3.91)		(0.08-0.61)

*PR,* Prevalence ratio.

*CI*, Confidence interval.

^†^ Maximum N for each category, which may vary between variables due to missing data.

* χ^2^ test of heterogeneity.

** χ^2^ test for linear trend.

With respect to socioeconomic status, maternal schooling and the household goods index were inversely related to the prevalence of underweight and stunting (Table 2). The prevalence ratio of stunting was 4.22 (CI 95%: 2.96-6.01) times higher among children whose mothers never frequented at least one full year of school. Children living in households with no regular source of income showed slightly higher prevalence rates of underweight and stunting. On the other hand, wasting was not associated with any of the three measures of socioeconomic status (Table 2).

**Table 2:** Prevalence of underweight, stunting, and wasting in indigenous children < 60 months, by socioeconomic variables, First National Survey of Indigenous People’s Health and Nutrition, Brazil, 2008-2009

	**N**^**†**^	**Underweight**		**Stunting**		**Wasting**	
		**Prevalence**	**PR**	**Prevalence**	**PR**	**Prevalence**	**PR**
		**(%)**	**(CI 95%)**	**(%)**	**(CI 95%)**	**(%)**	**(CI 95%)**
**Household goods index**			p < 0.001 **		p < 0.001 **		p = 0.25 **
1st tertile	2361	8.8	2.50	35.6	2.30	1.7	1.46
			(1.68-3.73)		(1.78-2.96)		(0.78-2.72)
2nd tertile	2123	5.2	1.46	25.3	1.64	1.0	0.87
			(1.12-1.89)		(1.36-1.97)		(0.43-1.76)
3rd tertile	1571	3.5	1.00	15.5	1.00	1.2	1.00
			(reference)		(reference)		(reference)
**Regular income**			p = 0.02 *		p = 0.003 *		p = 0.67 *
No	3535	6.5	1.33	27.9	1.23	1.3	1.10
			(1.04-1.69)		(1.07-1.42)		(0.70-1.74)
Yes	2504	4.9	1.00	22.7	1.00	1.2	1.00
			(reference)		(reference)		(reference)
**Maternal schooling**			p < 0.001 **		p < 0.001 **		p = 0.30 **
0 years	1058	10.5	3.68	39.9	4.22	1.9	1.19
			(1.92-7.06)		(2.96-6.01)		(0.48-2.91)
1-4 years	2634	6.7	2.33	29.9	3.16	1.3	0.79
			(1.43-3.82)		(2.33-4.27)		(0.40-1.57)
5-9 years	1521	3.3	1.16	18.1	1.92	0.8	0.50
			(0.69-1.95)		(1.49-2.46)		(0.17-1.44)
≥ 10 years	783	2.9	1.00	9.5	1.00	1.6	1.00
			(reference)		(reference)		(reference)

*PR*, Prevalence ratio.

*CI,* Confidence interval.

^†^ Maximum N for each category, which may vary between variables due to missing data.

* χ^2^ test of heterogeneity.

** χ^2^ test for linear trend.

Underweight and stunting were also associated with access to sanitation services. Children living in houses without piped drinking water, indoor sanitation facility (e.g., bathroom), or access to trash collection service showed a higher prevalence of underweight and stunting (Table 3).

**Table 3:** Prevalence rates of underweight, stunting, and wasting in indigenous children < 60 months according to characteristics of the household and sanitation, First National Survey of Indigenous People’s Health and Nutrition, Brazil, 2008-2009

	**N**^**†**^	**Underweight**		**Stunting**		**Wasting**	
		**Prevalence**	**PR**	**Prevalence**	**PR**	**Prevalence**	**PR**
		**(%)**	**(CI 95%)**	**(%)**	**(CI 95%)**	**(%)**	**(CI 95%)**
**Piped drinking water**			p < 0.001 *		p = 0.003 *		p = 0.08 *
Inside home	902	2.4	1.00	17.6	1.00	0.8	1.00
			(reference)		(reference)		(reference)
Outside home	2961	5.0	2.08	24.9	1.41	1.3	1.58
			(1.29-3.36)		(0.93-2.14)		(0.62-4.03)
Other	2180	10.3	4.28	33.3	1.89	1.7	2.07
			(2.57-7.12)		(1.20-2.96)		(0.84-5.09)
**Location used for defecation**			p = 0.001 *		p < 0.001 *		p = 0.01 *
Indoor household facility	3243	4.2	1.00	20.5	1.00	1.00	1.00
			(reference)		(reference)		(reference)
Outdoor household facility	680	6.5	1.53	34.6	1.68	0.7	0.71
			(1.04-2.26)		(1.31-2.16)		(0.32-1.60)
Other	2108	8.6	2.04	32.4	1.58	1.9	1.92
			(1.33-3.11)		(1.23-2.02)		(1.19-3.09)
**Trash collection service in the village**			p < 0.001 *		p < 0.001 *		p = 0.97 *
Yes	718	2.4	1.00	11.1	1.00	1.3	1.00
			(reference)		(reference)		(reference)
No	5313	6.6	2.74	28.4	2.55	1.3	0.96
			(1.85-4.06)		(1.59-4.10)		(0.51-1.82)

*PR,* Prevalence ratio.

*CI*, Confidence interval.

^†^ Maximum N for each category, which may vary between variables due to missing data.

* χ^2^ test of heterogeneity.

** χ^2^ test for linear trend.

Table 4 shows that maternal anemia was positively associated with underweight and stunting, whereas no clear pattern was observed for wasting. Birthweight was negatively associated with prevalence of underweight, wasting, and stunting. Low birthweight subjects were 10.0 (CI 95%: 5.67-17.5) times more likely to present underweight. Severe morbidity in the prior 12 months, as indicated by hospitalization, was also associated with underweight and stunting.

**Table 4:** Prevalence rates of underweight, stunting, and wasting in indigenous children < 60 months, according to maternal and child characteristics, First National Survey of Indigenous People’s Health and Nutrition, Brazil, 2008-2009

	**N**^**†**^	**Underweight**		**Stunting**		**Wasting**	
		**Prevalence**	**PR**	**Prevalence**	**PR**	**Prevalence**	**PR**
		**(%)**	**(CI 95%)**	**(%)**	**(CI 95%)**	**(%)**	**(CI 95%)**
**Maternal age (years)**			p = 0.66 **		p = 0.08 **		p = 0.06 **
< 20	814	5.2	1.00	26.6	1.00	2.5	1.00
			(reference)		(reference)		(reference)
20-29	3097	5.7	1.08	24.5	0.92	1.1	0.43
			(0.72-1.64)		(0.79-1.08)		(0.23-0.83)
30-39	1673	6.6	1.27	25.8	0.97	1.1	0.46
			(0.81-1.99)		(0.79-1.20)		(0.19-1.08)
≥ 40	455	6.0	1.15	31.3	1.18	1.1	0.47
			(0.71-1.87)		(0.96-1.45)		(0.16-1.36)
**Maternal anemia**			p = 0.006 *		p < 0.001 *		p = 0.15 *
No	3638	5.1	1.00	23.1	1.00	1.2	1.00
			(reference)		(reference)		(reference)
Moderate	2081	6.7	1.32	28.2	1.22	1.6	1.34
			(1.01-1.72)		(1.07-1.38)		(0.96-1.86)
Severe	223	12.2	2.38	44.2	1.91	0.7	0.61
			(1.08-5.24)		(1.46-2.50)		(0.14-2.66)
**Birthweight (grams)**			p < 0.001 **		p < 0.001 **		p < 0.001 **
< 2500	298	17.5	10.0	47.2	3.59	3.3	5.80
			(5.67-17.5)		(2.78-4.63)		(1.96-17.15)
2500-2999	935	8.9	5.06	32.3	2.45	2.1	3.77
			(2.83-9.06)		(1.93-3.10)		(1.37-10.34)
3000-3499	1607	4.2	2.37	19.9	1.51	1.1	1.91
			(1.38-4.06)		(1.18-1.94)		(0.68-5.39)
≥ 3500	1115	1.8	1.00	13.2	1.00	0.6	1.00
			(reference)		(reference)		(reference)
**Hospitalization in the prior 12 months**			p = 0.001*		p < 0.001 *		p = 0.59 *
No	4895	5.1	1.00	23.7	1.00	1.2	1.00
			(reference)		(reference)		(reference)
Yes	1119	9.0	1.78	33.8	1.43	1.5	1.27
			(1.27-2.48)		(1.22-1.67)		(0.53-3.08)

*PR,* Prevalence ratio.

*CI*, Confidence interval.

^†^ Maximum N for each category, which may vary between variables due to missing data.

* χ^2^ test of heterogeneity.

** χ^2^ test for linear trend.

**Figure 1:**
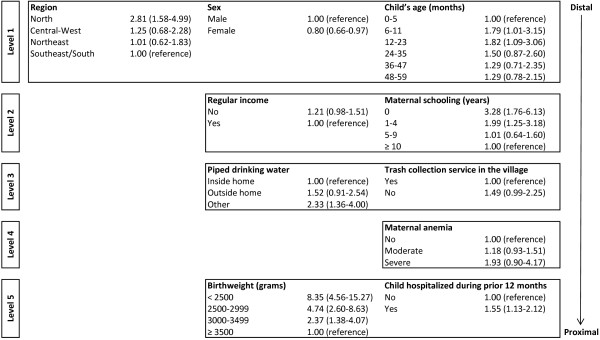
Hierarchical model for underweight among children < 5 years of age, First National Survey of Indigenous People’s Health and Nutrition, Brazil, 2008–2009.

In the multivariate model, children in the North region presented a higher prevalence of underweight, even after adjusting for child’s sex and age (Figure 1). In the second level, after controlling for the presence of regular household sources of income, region, sex, and child’s age, maternal schooling remained inversely related to prevalence of underweight. Children whose mothers did not frequent at least one year of school were 3.28 (CI 95%: 1.76-6.13) times more likely be underweight than those whose mothers had frequented at least 10 years of schooling. With respect to household environment characteristics, after controlling for the variables in the higher hierarchical level and source of drinking water, the effect of the presence of trash collection service in the community decreased from 2.74 to 1.49 and the confidence interval barely included the unity (1), although the variable still reached the significance level for retention in the model. Similarly, the effect of the source of drinking water used by members of the household was reduced in the multivariate analysis, although children without access to piped drinking water inside or outside the household still had a higher prevalence of underweight. Even after controlling for confounding by socioeconomic measures, demographic characteristics, household environmental conditions, maternal anemia and reported hospitalization in the prior 12 months, children whose birthweight was < 2,500 g were 8.35 (CI 95%: 4.56-15.27) times more likely to be underweight than those ≥ 3,500 (Figure 1).

**Figure 2:**
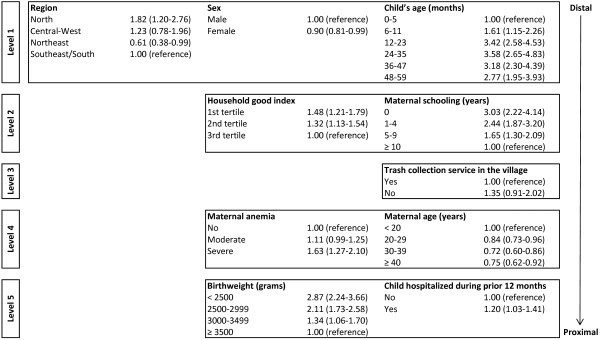
Hierarchical model for stunting among children < 5 years of age, First National Survey ff Indigenous People’s Health and Nutrition, Brazil, 2008–2009.

As shown in Figure 2, after adjusting for the variables in the first hierarchical level and maternal schooling, the prevalence of stunting among children living in households in the first tertile of the household good index decreased from 2.30 to 1.48 (CI 95%: 1.21-1.79), but the confidence interval did not include the unity. For maternal schooling the decrease in the magnitude of the prevalence ratio was smaller and the effect was also statistically significant. In the multivariate model, none of the variables related to the household environment were associated with stunting. However, maternal anemia was associated with a higher prevalence of stunting and children whose mothers presented severe anemia were 1.63 (CI 95%: 1.27-2.10) times more likely to be stunted than those whose mothers did not present severe anemia. Also, maternal age, birthweight, and no hospitalization record in the prior 12 months were inversely related to the prevalence of stunting.

**Figure 3:**
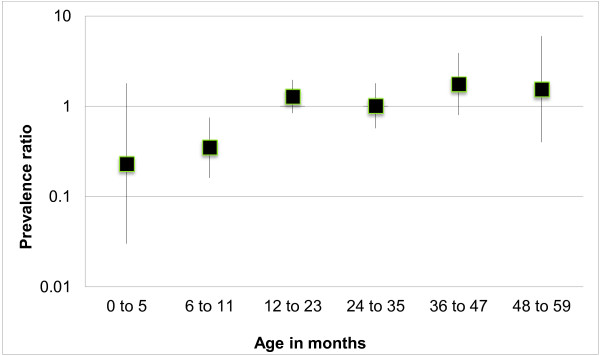
Prevalence ratio of underweight among children < 5 years of age who were breastfed as compared to those who were never breastfed, according to age categories, First National Survey of Indigenous People’s Health and Nutrition, Brazil, 2008–2009.

**Figure 4:**
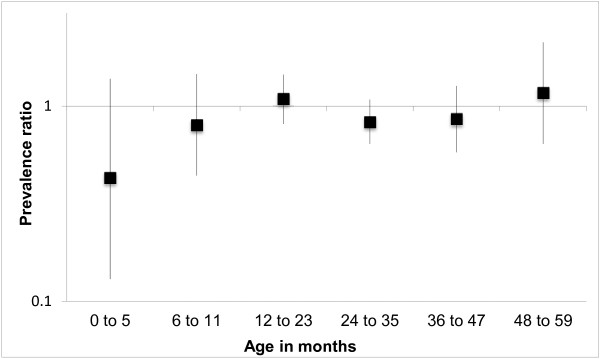
Prevalence ratio of stunting among children < 5 years of age who were breastfed as compared to those who were never breastfed, according to age categories, First National Survey of Indigenous People’s Health and Nutrition, Brazil, 2008–2009.

The effect of breastfeeding on underweight and stunting was evaluated for different child age categories. Figure 3 shows that among children aged < 12 months, the prevalence of underweight was lower for those who had never been breastfed. However, for children < 6 months the precision of estimates was low and the confidence interval included the unity. The protective effect of breastfeeding for underweight was not observed among children older than 1 year. Figure 4 shows that the protective effect of breastfeeding for stunting was observed only in the first 6 months of life, but the association was not statistically significant.

## Discussion

Relatively little information is available on the nutritional situation of indigenous children in Latin American countries. A recent compilation by the Pan American Health Organization of findings regarding the nutritional situation of indigenous children, based on the reference curves proposed by the World Health Organization [39], identified levels of undernutrition varying from 35 to 60% among Quechua and Aymara children in Bolivia and Peru, about 55% in indigenous children in Ecuador, and approximately 75% in indigenous children in Guatemala [44]. Thus, in many Latin American countries, rates of undernutrition among indigenous children tend to be much higher than those of non-indigenous children.

During the last two decades in Brazil there have been marked public policy improvements for indigenous peoples with implications for public health. For example, beginning in the 1990’s, the decennial national census included “indigenous” to the list of possible responses for the question on race or skin color [45]. During the same period, a distinct healthcare subsystem was implemented for indigenous peoples [46]. These policies aimed at improving the availability of health information, which demonstrated empirically that large inequalities exist between indigenous peoples and other segments of Brazilian society, particularly in relation to morbidity and mortality, which are part of a pernicious cycle marked by persistent poverty, exclusion, and disease.

Prior to the National Survey, the great majority of available information on the nutritional status of indigenous children in Brazil came from case studies carried out over the past two decades in specific communities and local populations, most located in Amazonia [14,22,23,47-50]. These case studies found levels of stunting well above those reported for non-indigenous children (see Leite et al. [51] for a review).

The results of the National Survey also reveal a very unfavorable nutritional scenario for indigenous children in the country. As compared to non-indigenous Brazilian children nationally, the present rate of stunting in indigenous children in Brazil (25.7%) is substantially higher than the current rate but comparatively close to that reported four decades ago [13,52]. The results of national household surveys since the 1970s show stunting (following the WHO growth curves [39]) in non-indigenous children < 5 years of age decreased sharply from 37.1% in 1974/1975 to 7.1% in 2006 [13].

Considered by major geopolitical region, the rates of stunting in indigenous children in Brazil ranged from two to five times higher than those observed for non-indigenous children. As reported by the Brazilian Ministry of Health [52], the rates of stunting among non-indigenous children in this age group were 5.5% in the Central-West, 5.6% in the Southeast, 5.8% in the Northeast, 8.5% in the South, and 14.7% in the North, the latter region presenting the highest index of poverty and the worse indicators of general health in the country.

A combination of factors is likely to be acting to increase the prevalence of undernutrition among indigenous children in Brazil. Stunting and underweight are closely related to chronic exposure to unfavorable socioeconomic and environmental conditions, poor energy and nutrient intake, and recurrent infectious and parasitic disease [53,54]. Previous studies have explained unfavorable rates of undernutrition among indigenous peoples in Brazil in terms of the impacts of increased participation in the market economy, reduced access to natural resources and land, sedentarization, and increased environmental contamination due to poor sanitary conditions, among other factors [14,20,55-58]. Consistent with these interpretations, in the multivariate analysis, we observed that such social determinants as household environmental characteristics, hospitalization, and socioeconomic condition were associated with higher prevalence of stunting and underweight.

Alternative explanations for the elevated prevalence rates of low height-for-age observed among indigenous children may be found in the human biology literature. For example, some lines of investigation draw on genetic-evolutionary hypotheses that populations in tropical forested environments tend to present smaller adult body size as an adaptive response to alleged environmental pressures (e.g., food restriction and high temperatures and humidity) [59,60]. According to such perspectives, the standard growth reference curves used in our analyses would not be applicable to some ethnic populations (such as indigenous peoples in the Amazon region of Brazil) due to a distinct genetic growth potential. However, in the field of public health nutrition, the relative weight of genetics is considered to be greater on final height achieved at the end of the growth period than on growth rates during infancy. Until at least the seventh year of life, human growth potential is essentially uniform worldwide, independent of region or ethnic group. During this phase of life, environmental factors (e.g., living conditions, sanitation, socioeconomic and education levels, diet, food security, coinfection, and access to health services) are considered to play a more dominant role in determining the distinct growth achievements observed among children in different populations [61-66]. It is now widely accepted that undernutrition is a multifaceted condition that cannot be fully understood on the basis of its immediate biological determinants – socioeconomic and ethnic disparities are at the root of the problem, particularly in countries that show sharp inequalities with regards to income, education, and access to health care [2,67-71].

Our data show an increase in the prevalence of stunting in the first 3 years of life. Indeed, among children less than 6 months of age the prevalence was 9.2%, whereas for those aged 24–35 months the prevalence was 32.7%. A small decrease in the prevalence of stunting was observed among children older than 36 months. These data suggests that there is no cohort effect in the studied population. Such increments in the prevalence of stunting reported in case studies of South American indigenous peoples were found to be strongly related to poor environmental conditions and weaning practices [14-17].

We observed substantially higher prevalence rates of stunting and underweight in the North region of the country as compared with all other regions. After controlling for maternal age, maternal schooling, the household goods index, presence of trash collection service in the village, maternal anemia, and birth weight, the prevalence ratio of stunting among children living in the North decreased from 1.83 to 1.58 (CI 95%: 1.07-2.34), whereas for underweight the prevalence ratio decreased from 2.87 to 1.91 (CI 95%: 1.04-3.51). These results demonstrate the close relationship between the higher rates of undernutrition observed in the North region and socioeconomic conditions, which have undergone major changes for indigenous populations in recent decades due to the rapid pace of economic development and environmental transformation in the region.

As we reported previously [20], the findings of the National Survey highlight major gaps in the availability of public services to indigenous villages in Brazil, such as education, basic sanitation, safe drinking water, and solid waste management. These are conditions that favor the occurrence of high levels of undernutrition in children, as was observed in the present study. With regard to the management of human waste, the most typical infrastructure observed was that of a simple pit latrine, with sewage rarely being collected or receiving any kind of treatment. Even in more developed regions of the country, such as the South/Southeast, nearly 40% of households in the sample reported defecating in the open. Only 5.9% of the households reported possessing any kind of sewage system. The management of household waste was also found to be precarious, with trash most commonly being discarded, burned, or buried in the peridomicile or elsewhere in the village.

Unfortunately, considering these inadequate sanitary conditions, it is unsurprising that children also present elevated levels of morbidity due to infectious and parasitic diseases. As previously reported, the National Survey found the proportion of reported hospitalizations of children during the prior 12 months to be elevated, with 19.3% of children being hospitalized during this period [20]. Diarrhea and respiratory infection were frequent causes of hospitalization. With respect to referred morbidity during the prior week, about one in four children (23.6%) presented diarrhea. Additionally, 51.2% of indigenous children nationally were found to be anemic. The health scenario outlined here for indigenous children facilitates the interaction between undernutrition and infection, widely described and characterized in the literature as cyclical and mutually reinforcing, not only because undernutrition contributes to increasing the severity and duration of infection, but also because recurrent infections tend to worsen the nutritional status of children [72-75].

Among infants younger than six months, those who were breastfed were less likely to be underweight or stunted, but the confidence intervals included the unity. Because breastfeeding was almost universal among infants younger than 6 months, with only 5.2% of infants in this age group not being breastfed at the time of the interview, the precision of the estimate on the effect of breastfeeding in this age group was low. On the other hand, breastfeeding did not protect children older than 12 months against stunting or underweight. Among older children, the benefits of breastfeeding may be overwhelmed by weaning foods with lower energy and nutrient content or contamination with microorganisms in situations of poverty or inadequate sanitation [76].

### Final considerations

The results presented here regarding the nutritional status of indigenous children from the First National Survey of Indigenous People’s Health and Nutrition in Brazil reveal striking health inequities involving a diverse set of socioeconomic and environmental factors. High prevalence rates of undernutrition were shown to be associated with socioeconomic variables including income, household goods, schooling, and access to sanitation services. They were also shown to be associated with breastfeeding, which is a highly cultural dimension of child dietary practices among many indigenous societies. Whereas the National Survey was the first study to address child nutrition among the indigenous peoples in Brazil on a national scale, providing important baseline data for future comparison, these findings further suggest the relevance of social, economic, and environmental factors at different scales (local, regional, and national) for the nutritional status of indigenous peoples.

Although the Brazilian Unified Health System (Sistema Único de Saúde – SUS) prioritizes the promotion of social and economic equity as part of public health research and promotion formulations, the findings reported here indicate that the full potential benefits of this policy orientation are not yet observable in the health and nutrition profile of the indigenous child population at a national scale. The worrying nutritional health profile of indigenous children in Brazil underscores the need for greater attention to this population segment by the Brazilian government.

Food and nutrition policies and interventions designed for indigenous peoples in Brazil must be tailored for consonance with the cultural lifestyles and food perceptions of target communities, going beyond the generalized distribution of energy-rich food items, typical of both governmental and non-governmental food relief initiatives. Measures aimed at improving childhood nutrition may potentially have immediate results, particularly with regards to stimulating child weight gain and improving child survival. Any intervention aimed at indigenous peoples in Brazil, however, must take into consideration this country’s enormous sociocultural diversity, with as many as 300 indigenous ethnic groups and over 200 indigenous languages living in diverse environmental settings. With so many distinctive societies within the Brazilian borders, it is all the more important to implement public health policies and measures aimed at reducing child undernutrition that incorporate sociocultural, economic, environmental, and biomedical dimensions of the problem (see Caldas and Santos [77] for a recent review of the development of nutrition policies aimed at indigenous peoples in Brazil).

Public policies must also address the need for more research on factors that remain understudied in indigenous populations despite being considered important underlying determinants of child nutritional status in accordance with the United Nations Children’s Fund’s framework for the causes of undernutrition [78,79]. For instance, research into cultural factors influencing childcare practices, including breastfeeding, weaning, and pregnancy, has the potential to help disentangle many of the complexities subsumed by standardly used variables that do not have uniform meaning in cross-cultural contexts, such as income, wealth, and maternal education.

## Abbreviations

ABRASCO: Associação Brasileira de Saúde Coletiva; CI: Confidence interval; CONEP: Comissão Nacional de Ética em Pesquisa; FUNAI: Fundação Nacional do Índio; FUNASA: Fundação Nacional de Saúde; PR: Prevalence ratio.

## Competing interests

The authors declare that they have no competing interests.

## Authors’ contributions

BLH, RVS, JRW, AMC, AMOA, PCIL, and CEAC formulated the research concept and design. All authors were involved in data collection. BLH and JVS conducted the statistical analyses. BLH, RVS, JRW, AMC, and CEAC wrote the manuscript and all other authors read and commented on the paper. The final version submitted for publication was read and approved by all authors.

## References

[B1] Black RE Allen LH Bhutta ZA Caulfield LE de Onis M Ezzati M Mathers C Rivera J Maternal and Child Undernutrition Study Group Maternal and child undernutrition 1 - maternal and child undernutrition: global and regional exposures and health consequences Lancet 2008 371 243 260 10.1016/S0140-6736(07)61690-0 18207566

[B2] Peña M Bacallao J Malnutrition and poverty Annu Rev Nutr 2002 22 241 253 10.1146/annurev.nutr.22.120701.141104 12055345

[B3] Torres C La equidad en materia de salud vista con enfoque étnico Rev Panam Salud Publica 2001 10 188 201 10.1590/S1020-49892001000900015 11702374

[B4] Montenegro RA Stephens C Indigenous health in Latin America and the Caribbean Lancet 2006 367 1859 1869 10.1016/S0140-6736(06)68808-9 16753489

[B5] Hotez PJ Neglected infections of poverty among the indigenous peoples of the Arctic PLoS Negl Trop Dis 2010 4 e606 10.1371/journal.pntd.0000606 20126272 PMC2811175

[B6] Larrea C Freire W Social inequality and child malnutrition in four Andean countries Rev Panam Salud Publica 2002 11 356 364 10.1590/S1020-49892002000500010 12162832

[B7] Bustos P Munoz S Vargas C Amigo H Evolution of the nutritional situation of indigenous and non-indigenous Chilean schoolchildren Ann Hum Biol 2009 36 298 307 10.1080/03014460902729536 19296262

[B8] Wilson WM Bulkan J Piperata BA Hicks K Ehlers P Nutritional status of Makushi Amerindian children and adolescents of Guyana Ann Hum Biol 2011 38 615 629 10.3109/03014460.2011.588248 21675938

[B9] Rosique J Restrepo MTC Manjarrés LMC Gálvez AA Santa JM Estado nutricional y hábitos alimentarios en indígenas Embera de Colombia Rev Chil Nutr 2010 37 270 280

[B10] Oyhenart EE Techenski MF Orden AB Nutritional status in two Mbyá-Guaraní communities from Misiones (Argentina) Homo 2003 54 170 179 10.1078/0018-442X-00069 14740367

[B11] Restrepo BN Estado nutricional de niños y niñas indígenas de hasta seis años de edad en el resguardo Embera-Katío, Tierralta, Córdoba, Colombia Biomedica 2006 26 517 527 17315478

[B12] Benefice E Monroy SL Jiménez S López R Nutritional status of Amerindian children from the Beni River (lowland Bolivia) as related to environmental, maternal and dietary factors Public Health Nutr 2006 9 327 335 10.1079/PHN2006852 16684384

[B13] Monteiro CA Benício MHD Conde WL Konno S Lovadino AL Barros AJD Victora CG Narrowing socioeconomic inequality in child stunting: the Brazilian experience, 1974–2007 Bull World Health Organ 2010 88 305 311 10.2471/BLT.09.069195 20431795 PMC2855601

[B14] Ferreira AA Welch JR Santos RV Gugelmin SA Coimbra CEA Jr Nutritional status and growth of indigenous Xavante children, Central Brazil Nutr J 2012 11 3 10.1186/1475-2891-11-3 22236407 PMC3317817

[B15] Orellana JDY Coimbra CEA Jr Lourenço AEP Santos RV Estado nutricional e anemia em crianças Suruí, Amazônia, Brasil J Pediatr (Rio J) 2006 82 383 388 10.1590/S0021-75572006000600013 17003939

[B16] Picoli RP Carandina L Ribas DL Estado nutricional e fatores associados à estatura de crianças da Terra Indígena Guarita, Sul do Brasil Cad Saude Publica 2006 22 223 227 10.1590/S0102-311X2006000100025 16501752

[B17] Leite MS Santos RV Coimbra CEA Jr Sazonalidade e estado nutricional de populações indígenas: o caso Wari’, Rondônia, Brasil Cad Saude Publica 2007 23 2631 2642 10.1590/S0102-311X2007001100011 17952256

[B18] Menegolla IA Drachler ML Rodrigues IH Schwingel LR Scapinello E Pedroso MB Leite JCC Estado nutricional e fatores associados à estatura de crianças da Terra Indígena Guarita, Sul do Brasil Cad Saude Publica 2006 22 395 406 10.1590/S0102-311X2006000200017 16501752

[B19] Morais MB Alves GMS Fagundes-Neto U Estado nutricional de crianças índias terenas: evolução do peso e estatura e prevalência atual de anemia J Pediatr (Rio J) 2005 81 383 389 10.2223/JPED.1389 16247540

[B20] Coimbra CEA Jr Santos RV Welch JR Cardoso AM Souza MC Garnelo L Rassi E Follér M-L Horta BL The First National Survey of Indigenous People’s Health and Nutrition in Brazil: rationale, methodology, and overview of results BMC Publ Health 2013 13 52 10.1186/1471-2458-13-52 PMC3626720 23331985

[B21] Santos RV Crescimento físico e estado nutricional de populações indígenas brasileiras Cad Saude Publica 1993 9 Suppl 1 46 57 15448820

[B22] Orellana JDY Santos RV Coimbra CEA Jr Leite MS Avaliação antropométrica de crianças indígenas menores de 60 meses, a partir do uso comparativo das curvas de crescimento NCHS/1977 e OMS/2005 J Pediatr (Rio J) 2009 85 117 121 10.1590/S0021-7557200900020000619225686

[B23] Escobar AL Santos RV Coimbra CEA Jr Avaliação nutricional de crianças indígenas Pakaánova (Wari’), Rondônia, Brasil Revista Brasileira de Saúde Materno Infantil 2003 3 457 461 10.1590/S1519-38292003000400010

[B24] Mondini L Rodrigues D Gimeno SGA Baruzzi RG Estado nutricional e níveis de hemoglobina em crianças Aruak e Karibe: povos indígenas do Alto Xingu, Brasil Central, 2001–2002 Rev Bras Epidemiol 2009 12 469 477 10.1590/S1415-790X2009000300015

[B25] San Sebastián M Hurtig A-K Review of health research on indigenous populations in Latin America, 1995–2004 Salud Publica Mex 2007 49 316 320 10.1590/S0036-36342007000400012 17710281

[B26] Garnelo L Brandão LC Levino A Dimensões e potencialidades dos sistemas de informação geográfica na saúde indígena Rev Saude Publica 2005 39 634 640 10.1590/S0034-89102005000400018 16113915

[B27] Souza LG Santos RV Carvalho MS Pagliaro H Flowers NM Coimbra CEA Jr Demography and health of the Xavante Indians from Central Brazil Cad Saude Publica 2011 27 1891 1905 10.1590/S0102-311X2011001000003 22031194

[B28] Cardoso AM Santos RV Coimbra CEA Jr Mortalidade infantil segundo raça/cor no Brasil: o que dizem os sistemas nacionais de informação? Cad Saude Publica 2005 21 1602 1608 10.1590/S0102-311X2005000500035 16158168

[B29] Victora CG Adair L Fall C Hallal PC Martorell R Richter L Sachdev HS Maternal and Child Undernutrition Study Group Maternal and child undernutrition: consequences for adult health and human capital Lancet 2008 371 340 357 10.1016/S0140-6736(07)61692-4 18206223 PMC2258311

[B30] Horta BL Sibbritt DW Lima RC Victora CG Weight catch-up and achieved schooling at 18 years of age in Brazilian males Eur J Clin Nutr 2009 63 369 374 10.1038/sj.ejcn.1602934 17957192

[B31] Horta BL Gigante DP Osmond C Barros FC Victora CG Intergenerational effect of weight gain in childhood on offspring birthweight Int J Epidemiol 2009 38 724 732 10.1093/ije/dyp168 19376883 PMC2689398

[B32] Ramakrishnan U Martorell R Schroeder DG Flores R Role of intergenerational effects on linear growth J Nutr 1999 129 544S 549S 10064328 10.1093/jn/129.2.544S

[B33] Klebanoff MA Graubard BI Kessel SS Berendes HW Low-birth-weight across generations JAMA 1984 252 2423 2427 10.1001/jama.1984.03350170025013 6481929

[B34] Guimarães FMS Divisão Regional do Brasil 1942 Rio de Janeiro: Instituto Brasileiro de Geografia e Estatística

[B35] Lemeshow S Hosmer D Klar J Lwanga SK Adequacy of Sample Size in Health Studies 1990 Chichester, England: John Wiley & Sons

[B36] Ohlsson E Sequential poisson sampling J Offic Stat 1998 14 149 162

[B37] Davies PSW Roodveldt R Marks G Standard Methods for the Collection and Collation of Anthropometric Data in Children 2001 Canberra: National Food and Nutrition Monitoring and Surveillance Project, The Commonwealth Department of Health and Aged Care, Australia

[B38] WHO - World Health Organization Physical Status: the Use and Interpretation of Anthropometric Indicators of Nutritional Status 1995 Geneve: WHO

[B39] de Onis M Onyango AW WHO child growth standards Lancet 2008 371 204 204 10.1016/S0140-6736(08)60131-2 18207015

[B40] WHO - World Health Organization Length/Height-for-Age, Weight-for-Age, Weight-for-Length, Weight-for-Height and Body Mass Index-for-Age: Methods and Development 2006 Geneve: WHO

[B41] WHO - World Health Organization Iron Deficiency Anaemia Assessment, Prevention and Control. A Guide for Programme Managers 2001 Geneva: World Health Organization

[B42] Barros AJ Hirakata V Alternatives for logistic regression in cross-sectional studies: an empirical comparison of models that directly estimate the prevalence ratio BMC Med Res Methodol 2003 3 21 10.1186/1471-2288-3-21 14567763 PMC521200

[B43] Victora CG Huttly SR Fuchs SC Olinto MTA The role of conceptual frameworks in epidemiological analysis: a hierarchical approach Int J Epidemiol 1997 26 224 227 10.1093/ije/26.1.224 9126524

[B44] Lutter CK Chaparro CM Malnutrition in Infants and Young Children in Latin America and the Caribbean: Achieving the Millenium Development Goals 2008 Washington, D.C.: The Pan American Health Organization

[B45] Pereira NOM Santos RV Azevedo MM Pagliaro H, Azevedo MM, Santos RV Perfil demográfico e socioeconômico das pessoas que se autodeclararam ‘indígenas’ nos censos demográficos de 1991 e 2000 Demografia dos Povos Indígenas no Brasil 2005 Rio de Janeiro: Editora Fiocruz 155 166

[B46] Santos RV Cardoso AM Garnelo L Coimbra CEA Jr Chaves MBG Giovanella L, Escorel S, Lobato LVC, Noronha JC, Carvalho AI Saúde dos povos indígenas e políticas públicas no Brasil Políticas e Sistema de Saúde no Brasil 2008 Rio de Janeiro: Editora Fiocruz 1035 1056

[B47] Morais MB Fagundes-Neto U Mattos AP Baruzzi RG Estado nutricional de crianças índias do Alto Xingu em 1980 e 1992 e evolução pondero-estatural entre o primeiro e o quarto anos de vida Cad Saude Publica 2003 19 543 550 10.1590/S0102-311X2003000200021 12764470

[B48] Mattos A Morais MB Rodrigues DA Baruzzi RG Nutritional status and dietary habits of Indian children from Alto Xingu (Central Brazil) according to age J Am Coll Nutr 1999 18 88 94 10067664 10.1080/07315724.1999.10718832

[B49] Martins SJ Menezes RC Evolução do estado nutricional de menores de cinco anos em aldeias indígenas na tribo Parakanã, na Amazônia Oriental brasileira Rev Saude Publica 1994 28 1 8 10.1590/S0034-89101994000100001 7997819

[B50] Santos RV Coimbra CEA Jr Socioeconomic transition and physical growth of Tupí-Mondê Amerindian children of the Aripuanã Park, Brazilian Amazon Hum Biol 1991 63 795 819 1959911

[B51] Leite MS Santos RV Coimbra CEA Jr Gugelmin SA Kac G, Sichieri R, Gigante DP Alimentação e nutrição dos povos indígenas no Brasil Epidemiologia Nutricional 2007 Rio de Janeiro: Editora Fiocruz 503 518

[B52] MS-Ministério da Saúde Pesquisa Nacional de Demografia e Saúde da Criança e da Mulher – PNDS 2006: Dimensões do Processo Reprodutivo e da Saúde da Criança Série G Estatística e Informação em Saúde 2009 Brasília: Ministério da Saúde 302

[B53] Wamani H Astrom AN Peterson S Tumwine JK Tylleskar T Predictors of poor anthropometric status among children under 2 years of age in rural Uganda Public Health Nutr 2006 9 320 326 10.1079/PHN2006854 16684383

[B54] Adair LS Guilkey DK Age-specific determinants of stunting in Filipino children J Nutr 1997 127 314 320 9039833 10.1093/jn/127.2.314

[B55] Coimbra CEA Jr Flowers NM Salzano FM Santos RV The Xavánte in Transition: Health, Ecology, and Bioanthropology in Central Brazil 2002 Ann Arbor: University of Michigan Press

[B56] Coimbra CEA Jr Santos RV Salzano FM, Hurtado AM Emerging health needs and epidemiological research in indigenous peoples in Brazil Lost Paradises and the Ethics of Research and Publication 2004 Oxford: Oxford University Press 89 109

[B57] Lourenço AEP Santos RV Orellana JDY Coimbra CEA Jr Nutrition transition in Amazonia: obesity and socioeconomic change in the Suruí indians from Brazil Am J Hum Biol 2008 20 564 571 10.1002/ajhb.20781 18442078

[B58] Welch JR Ferreira AA Santos RV Gugelmin SA Werneck G Coimbra CEA Jr Nutrition transition, socioeconomic differentiation, and gender among adult Xavante Indians, Brazilian Amazon Hum Ecol 2009 37 13 26 10.1007/s10745-009-9216-7

[B59] Holmes R Sponsel LE Small is adaptive. Nutritional anthropology of native Amazonians Indigenous Peoples and the Future of Amazonia 1995 Tucson, AZ: University of Arizona Press 121 148

[B60] Stinson S Physical growth of Ecuadorian Chachi Amerindians Am J Hum Biol 1989 1 697 707 10.1002/ajhb.1310010607 28597482

[B61] Eveleth PB Tanner JM Worldwide Variation in Human Growth 1976 Cambridge: Cambridge University Press

[B62] Habicht J-P Yarbrough C Martorell R Malina RM Klein RE Height and weight standards for preschool children. How relevant are ethnic differences in growth potential? Lancet 1974 303 611 615 10.1016/S0140-6736(74)92663-4 4132271

[B63] Cameron N Human growth, nutrition, and health status in Sub-Saharan Africa Yearb Phys Anthropol 1991 34 211 250 10.1002/ajpa.1330340611 12343663

[B64] Petrou S Kupek E Poverty and childhood undernutrition in developing countries: a multi-national cohort study Soc Sci Med 2010 71 1366 1373 10.1016/j.socscimed.2010.06.038 20691525

[B65] Martorell R Young MF Patterns of stunting and wasting: potential explanatory factors Advances in Nutrition 2012 3 227 233 10.3945/an.111.001107 22516733 PMC3648726

[B66] Van de Poel E Hiosseinpoor AR Jehu-Appiah C Vega J Speybroeck N Malnutrition and the disproportional burden of the poor: the case of Ghana Int J Equity Health 2007 6 21 10.1186/1475-9276-6-21 18045499 PMC2245943

[B67] Kanjilal B Mazumdar PG Mukherjee M Rahman MH Nutritional status of children in India: household socio-economic condition as the contextual determinant Int J Equity Health 2010 9 19 10.1186/1475-9276-9-19 20701758 PMC2931515

[B68] Mwangome M Prentice A Plugge E Nweneka C Determinants of appropriate child health and nutrition practices among women in rural Gambia J Health Popul Nutr 2010 28 167 172 20411680 10.3329/jhpn.v28i2.4887 PMC2980879

[B69] Larrea C Kawachi I Does economic inequality affect child malnutrition? The case of Ecuador Soc Sci Med 2005 60 165 178 10.1016/j.socscimed.2004.04.024 15482876

[B70] Lee J Houser RF Must A Fulladolsa PP Bermudez OI Socioeconomic disparities and the familial coexistence of child stunting and maternal overweight in Guatemala Econ Hum Biol 2012 10 232 241 10.1016/j.ehb.2011.08.002 21889428 PMC3586429

[B71] Zere E McIntyre D Inequities in under-five child malnutrition in South Africa Int J Equity Health 2003 2 7 10.1186/1475-9276-2-7 14514357 PMC201028

[B72] Scrimshaw NS SanGiovanni JP Synergism of nutrition, infection, and immunity: an overview Am J Clin Nutr 1997 66 Suppl. 2 467 477 10.1093/ajcn/66.2.464S 9250134

[B73] Hall A Zhang Y MacArthur C Baker S The role of nutrition in integrated programs to control neglected tropical diseases BMC Med 2012 10 41 10.1186/1741-7015-10-41 22533927 PMC3378428

[B74] Keusch GT The history of nutrition: malnutrition, infection and immunity J Nutr 2003 133 Suppl 336S 340S 12514322 10.1093/jn/133.1.336S

[B75] Solomons NW Malnutrition and infection: an update Br J Nutr 2007 98 Suppl 1 S5 S10 17922960 10.1017/S0007114507832879

[B76] Rowland MGM Barrell RAE Whitehead RG Bacterial-contamination in traditional Gambian weaning foods Lancet 1978 1 136 138 87565 10.1016/s0140-6736(78)90432-4

[B77] Caldas ADR Santos RV Vigilância alimentar e nutricional para os povos indígenas no Brasil: Análise da construção de uma política pública em saúde Physis 2012 22 545 565 10.1590/S0103-73312012000200008

[B78] UNICEF - United Nations Children’s Fund Strategy for Improved Nutrition of Children and Women in Developing Countries 1990 New York: UNICEF 10.1007/BF02810402 1937618

[B79] Engle P Menon P Haddad L Care and nutrition: concepts and measurement World Dev 1999 27 1309 1337 10.1016/S0305-750X(99)00059-5

